# How well do individual first pass perfusion images correlate with fully quantitative myocardial blood flow pixel maps?

**DOI:** 10.1186/1532-429X-18-S1-P108

**Published:** 2016-01-27

**Authors:** Tendoh Timoh, Li-Yueh Hsu, Allison D Ta, Andrew E Arai

**Affiliations:** 1 Cardiology, Medstar Georgetown University Hospital and Washington Hospital Center, Washington, DC USA; 2 Advanced Cardiovascular Imaging Laboratory, National Heart, Lung, and Blood Institute, National Institutes of Health, Bethesda, MD USA

## Background

Quantitative pixel mapping in cardiac magnetic resonance (CMR) has been shown to be an accurate estimate of myocardial blood flow (MBF) in perfusion imaging when compared to a microsphere reference standard. We hypothesize that there is a time period during contrast enhancement when individual perfusion images correlate closely with MBF pixel maps.

## Methods

27 subjects (17 male; age 62 ± 11 years) with coronary artery disease as defined by quantitative coronary angiography were included in this study. Greater than 70% luminal coronary stenosis of the major epicardial coronaries was present in 2/3^rd^ of the subjects. Perfusion imaging was performed using a saturation recovery steady-state free precession dual-sequence method at 1.5 T during Regadenoson vasodilator stress and at rest, using 0.05 mmol/kg Gadolinium-DTPA. Pixel-wise MBF maps were calculated from a mid-ventricular motion-corrected image series. Myocardial regions of interest were manually traced on the perfusion image series to restrict analysis to the myocardium. The MBF pixel values were then correlated with individual perfusion image signal intensities on a pixel-by-pixel basis for each perfusion image in the series (Pearson's correlation coefficient).

## Results

For stress myocardial perfusion images, the average time from start of myocardial enhancement to peak myocardial signal intensity was 7.4 ± 2.2 seconds. The highest correlation between stress perfusion images and MBF pixel maps occurred at 4.5 ± 1.9 seconds after the start of myocardial enhancement and had an R value of 0.80 ± 0.08. The correlation remained within 5 percent of peak correlation for 2.8 ± 1.7 seconds.

**Figure 1:**
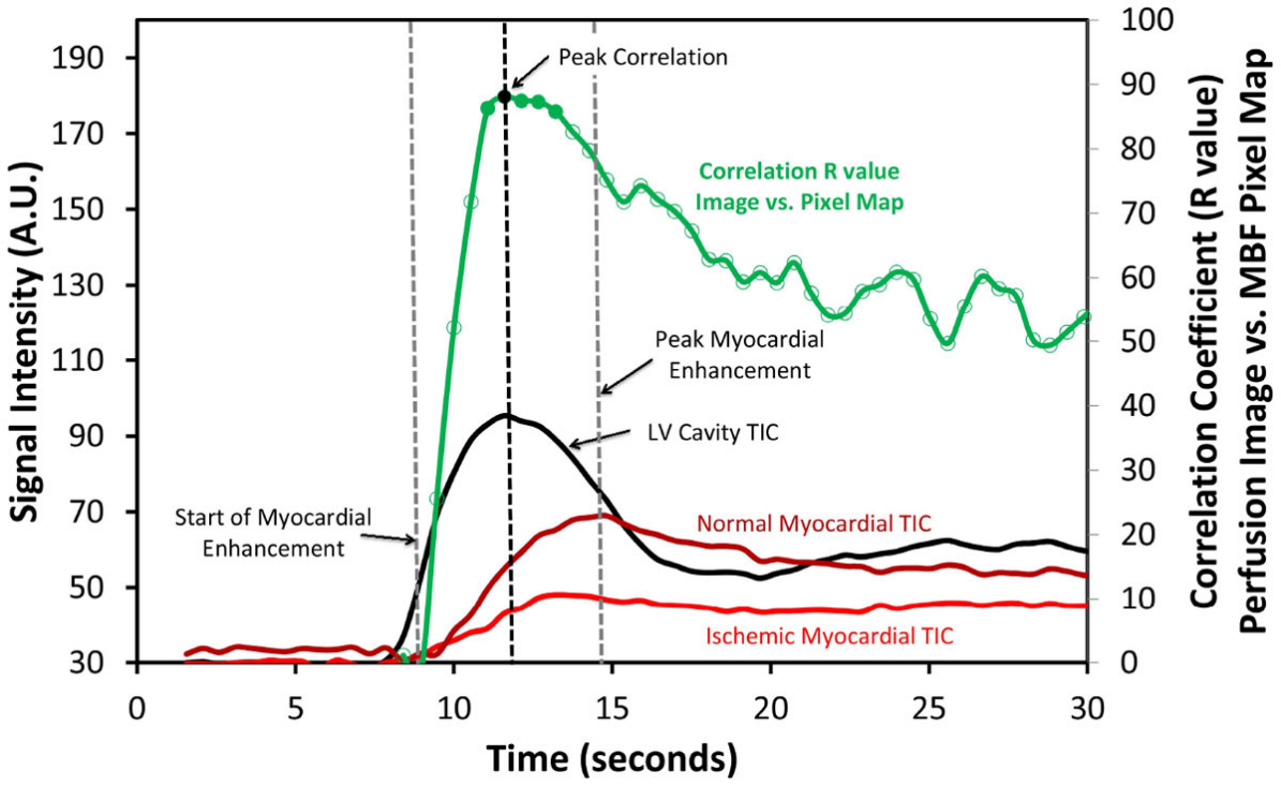
**Correlation between stress MBF Pixel Map and signal intensity on each individual stress perfusion image superimposed on LV and myocardial time intensity curves in a patient with severe left-anterior descending coronary artery disease**. The vasodilator response is significantly less pronounced in the ischemic segment corresponding to the diseased left-anterior descending coronary artery. The highest correlation tends to occur significantly prior to the time of peak myocardial enhancement. TIC: time intensity curve.

**Figure 2:**
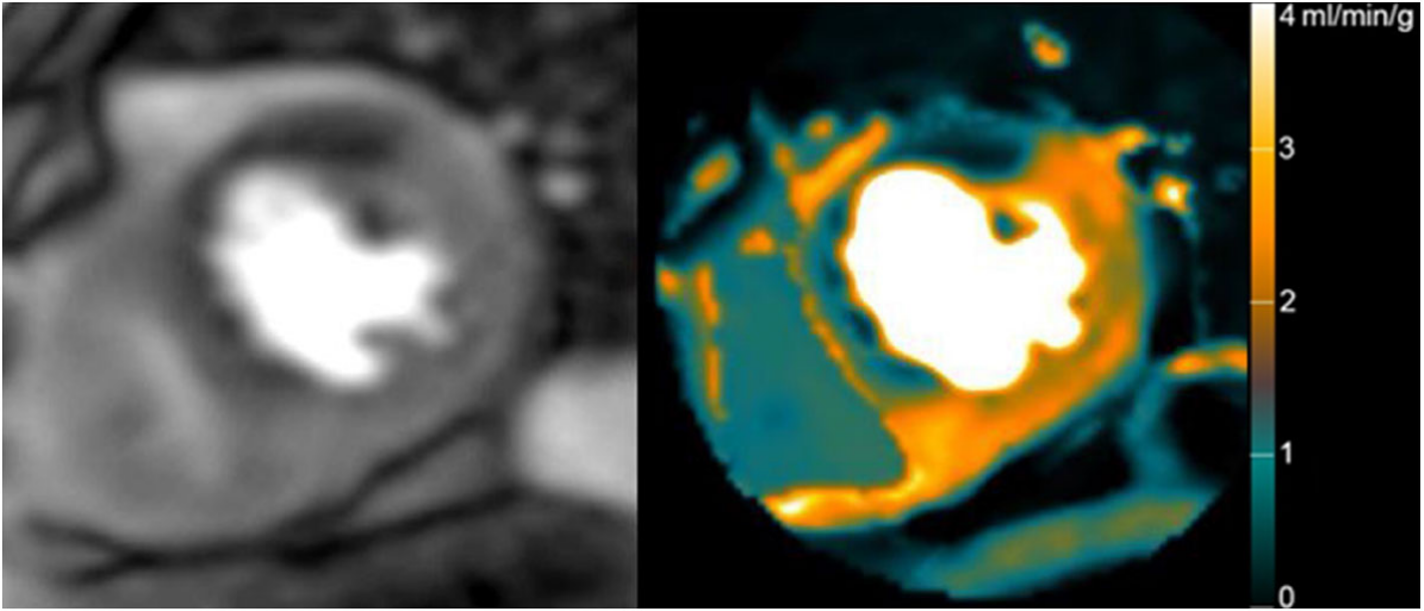
**CMR Stress Perfusion (on left) and Quantitative MBF Perfusion Pixel Map Image (on right) in the same patient with severe left-anterior descending coronary artery disease from Figure 1a**. The stress perfusion image is that acquired from the predicted time period of peak correlation. Note there is differentially reduced blood flow in the distribution of the diseased left-anterior descending coronary artery relative to remote myocardium. The range of low and high values is suitable for correlation statistics on a pixel-by-pixel basis.

For rest myocardial perfusion images, the average time from start of myocardial enhancement to peak myocardial signal intensity was 10.9 ± 3.2 seconds. The highest correlation between rest perfusion images and MBF pixel maps occurred at 7.5 ± 3.0 seconds after the start of myocardial enhancement and had an R value of 0.55 ± 0.16. The correlation remained within 5 percent of peak correlation for 2.9 ± 1.8 seconds. See Figure 1 and Figure 2.

## Conclusions

There is a characteristic and consistent time period during contrast enhancement on the perfusion images that correlate best to the quantitative MBF perfusion pixel maps. The correlation, as expected, is higher with stress perfusion imaging than rest perfusion imaging. It may be possible to target perfusion images in this time period to improve the efficiency of clinical image interpretation.

